# Identification of an Immunosuppressive Cell Population during Classical Swine Fever Virus Infection and Its Role in Viral Persistence in the Host

**DOI:** 10.3390/v11090822

**Published:** 2019-09-04

**Authors:** Jose Alejandro Bohorquez, Sara Muñoz-González, Marta Pérez-Simó, Concepción Revilla, Javier Domínguez, Llilianne Ganges

**Affiliations:** 1 OIE Reference Laboratory for classical swine fever, IRTA-CReSA, Campus Universitat de Barcelona, 08193 Bellaterra, Spain; 2 Dpto. Biotecnología, Instituto Nacional de Investigación y Tecnología Agraria y Alimentaria (INIA), 28040 Madrid, Spain

**Keywords:** CSFV, viral persistence, immunosuppression, peripheral blood, bone marrow cells, 6D10 cells, MDSC, interferon gamma

## Abstract

Classical swine fever virus (CSFV) remains a highly important pathogen, causing major losses in the swine industry. Persistent infection is highly relevant for CSFV maintenance in the field; however, this form of infection is not fully understood. An increase in the granulocyte population has been detected in CSFV persistently infected animals. The aim of this work was to evaluate the possible immunosuppressive role of these cells in CSFV persistent infection. The phenotype of peripheral blood and bone marrow cells from persistently infected and naïve animals was evaluated by flow cytometry, and the capacity of specific cell subsets to reduce the interferon gamma (IFN-γ) response against unspecific and specific antigen was determined using co-culture assays. The frequency of granulocytic cells was increased in cells from CSFV persistently infected pigs and they showed a phenotype similar to immunosuppressive cell populations found in persistent infection in humans. These cells from persistently infected animals were able to reduce the IFN-γ response against unspecific and specific antigen. Our results suggest that immature immunosuppressive cell populations play a role in CSFV persistent infection in swine. The information obtained by studying the role of myeloid derived suppressor cells (MDSC) during CSFV persistent infection may extrapolate to other viral persistent infections in mammals.

## 1. Introduction

Classical swine fever (CSF) remains a highly relevant disease in swine, causing major losses to the industry which are related to various forms of disease [1]. CSF has been eradicated in the US and Western Europe and remains endemic in several countries, including Asia, Central and South America, and Eastern Europe, with a recent outbreak being reported in Japan [2]. The disease is caused by the CSF virus (CSFV), a positive stranded RNA virus that belongs to the *Pestivirus* genus within the *Flaviviridae* family [3].

Several viruses have developed mechanisms to induce persistent infection and evade the immune response in the host, favouring viral prevalence. Examples of viral persistence can be found in humans and animals after infections with different viruses such as *Adenovirus*, *Herpesvirus*, *Retrovirus*, *Flavivirus*, *Hepadnavirus*, *Papillomavirus*, and *Pestivirus* among others [4,5,6,7,8,9,10,11]. In this regard, after infection in utero, CSFV can generate congenital persistent infection [1,11,12] similarly to other pestiviruses affecting ruminants, such as bovine viral diarrhoea virus (BVDV) and border disease virus (BDV) [13,14]. Likewise, it has also been reported that CSFV is able to cause postnatal persistent infection following infection with a moderate virulence strain in piglets from the day of birth until 21 days after [15,16], demonstrating the viral capacity to induce persistent infection in the host. Both congenitally and postnatally persistently infected animals are clinically healthy or develop clinical signs that are not commonly associated with CSF. These pigs develop high viraemia and shed a high virus level for a long period in the absence of a specific humoral response to the virus [15,16,17]. Interestingly, an immunosuppressed status in the peripheral blood mononuclear cells (PBMCs) from postnatal persistently infected animals has been demonstrated [15], which were measured for their inability to generate IFN-γ response against CSFV or a mitogen/polyclonal activator like phytohaemagglutinin (PHA) [15]. Additionally, high quantities of interleukin 10 (IL-10), a well-characterised immunosuppressive cytokine that inhibits a broad spectrum of immune responses [18], was detected after CSFV stimulation in PBMCs of persistently infected animals [15]. Notably, during the persistent infection phase with CSFV, the type I IFN response was impaired in the infected animals [17,19]. Moreover, the phenotypical profile in the bone marrow haematopoietic cells (BMHCs) from these animals showed an increase in the granulocytic (6D10^+^) cell population [20], being identified as a target for CSFV replication [15].

Myeloid derived suppressor cells (MDSC) are immature myeloid cell populations that are able to induce immune suppression [21]. First described in 1987 [22], MDSC were initially associated with immunosuppression in tumour microenvironments through multiple mechanisms, mainly IL-10, arginase 1, induced Nitric oxide synthases (iNOS), and reactive oxygen species [21,23]. More recently, MDSC have been implicated with viral infections such as Hepatitis C virus (HCV), Hepatitis B virus (HBV), and human immunodeficiency virus (HIV) [4,5,6]. In these infection models, the immunosuppression caused by MDSC populations has been suggested to facilitate the viral persistence and is also related to the worst clinical outcomes. So far, these cells have only been characterized in human and murine models, and in both species, there appears to be mainly two phenotypes, the monocytic-MDSC (M-MDSC) and the polymorphonuclear-MDSC (PMN-MDSC), which are morphologically and phenotypically similar to monocytes and neutrophils, respectively [21,23,24]. 

The characterization of the mechanisms used by MDSC to cause immunosuppression after viral infection has been one of the areas of interest in research as of late. However, the understanding of the interaction of these cells with the infected host in an in vivo model and their relation with the viral persistence in mammalian hosts remains a poorly studied area [21,24,25]. Considering this background, we hypothesized that the increase in the granulocyte precursor cell population during CSFV persistent infection might be an underlying factor in the impaired innate and adaptive immune response against CSFV in the infected host. Thus, we studied the role of the granulocyte precursor cell population as a MDSC population and its association with immunosuppression during CSFV persistent infection. The phenotypic profile of BMHC and PBMC from CSFV persistently infected animals was evaluated and multiple functional assays were performed in order to characterize the population associated with the immunosuppressive capacity that may promote viral persistence.

## 2. Materials and Methods

### 2.1. Cells and Viruses

The porcine kidney cell line PK-15 was obtained from ATCC (CCL-33, Middlesex, England) and were cultured in Dulbecco’s Modified Eagle Medium (DMEM), supplemented with 10% foetal bovine serum (FBS), Pestivirus-free, at 37 °C in 5% CO_2_.

The CSFV moderately virulent Catalonia 01 (Cat01) strain, which belongs to the 2.3 subgenotype and has been proven to induce postnatal persistent infection in newborn piglets, was used [15,26]. The Alfort 187 strain, genogroup 1.1, was used in the neutralisation peroxidase-linked assay (NPLA). Viruses were grown in the PK-15 cell line that were infected with 0.1 TCID_50_/cell in 2% FBS during 72 h of incubation. Peroxidase-linked assay (PLA) [27] was used for viral isolation and titration following the statistical methods previously described [28]. The attenuated vaccine (C-strain) belongs to the CSFV 1.1 genogroup and was used in Spain in the 1980s for CSF control. This vaccine has 100% homology with the Z46258 strain. This vaccine strain was employed to immunize animals in order to obtain IFN-γ producing PBMCs for the co-culture assays [29,30].

### 2.2. Experimental Infection

To reproduce the CSF persistent infection, an experimental infection in piglets was carried out according to previous studies [15]. Seven piglets (Group 1, numbered 1 to 7), born to a pestivirus-free sow in the biosecurity level 3 animal facility (BSL3) of the Centre de Recerca en Sanitat Animal (CReSA), were inoculated intranasally at 5 days of age with 2.5 × 10^4^ TCID of CSFV Cat01 strain. The inoculation of the piglets was conducted separately from the sow and the animals were housed with their mother until 28 days after birth. Afterwards, the piglets were fed a conventional piglet starter diet (Neopig, Kwikstart, CARGILL, Zaragoza, España) until their time of euthanasia according to previous studies conducted in CReSA [31,32].

A trained veterinarian recorded the rectal temperature and clinical signs daily in a blinded manner. Serum and rectal swab samples were collected every week after infection for five weeks. At this time, the piglets were euthanized and whole blood samples were collected in order to isolate peripheral blood mononuclear cells (PBMCs). During necropsy, the femurs and humeri of selected piglets (pigs 1, 2, 4, 5, and 6) were obtained in order to harvest bone marrow haematopoietic cells (BMHCs). The procedure for the euthanasia of the animals was based on an accepted method included in European Directive 2010/63/EU, using an anaesthetic overdose of 60–100 mg of pentobarbital per kilogram of weight, administered via the vena cava.

In addition, a whole blood sample was obtained from two donor animals (Group 2, numbered 8 and 9) from a vaccine trial [29]; these animals were 6 weeks of age and had been vaccinated with one pig dose (equivalent to 100 protective doses) of the C-strain vaccine. They were proven to have an efficient specific cellular immune response against CSFV, which was measured by ELISPOT assay at the time of sampling [29]. Finally, three naïve animals (Group 3, numbered 10 to 12) of the same origin and age were used as controls. Whole blood samples, as well as the femurs and humeri from these animals were collected at necropsy. The experiments were approved by the Ethics Committee for Animal Experiments of the Autonomous University of Barcelona (UAB), under number 8646, (31st July 2017) according to existing national and European regulations.

### 2.3. CSFV RNA and Antibody Detection

Sera and rectal swab samples from these animals were used for RNA extraction with the NucleoSpin RNA isolation kit (Macherey-Nagel, Düren, Germany), following the manufacturer’s instructions. The presence of CSFV RNA in the samples was detected by real time RT-qPCR [33]. Positive results were considered for threshold cycle values (CT) equal to or less than 42. Samples in which fluorescence was undetectable were considered negative. As previously described, Ct values from 10 to 22 were considered as high, from 23 to 28 were considered as moderate, and between 29 and 42 were considered as low RNA viral load [34]. The specific humoral immune response against CSFV was evaluated using a commercial ELISA kit (IDEXX, Liebfeld, Switzerland) detecting the presence of E2-specific antibodies in serum. According to the manufacturer’s instructions, the samples were considered as negative when the blocking percentage value was below 30% and positive when it was above 40%. Serum samples were also tested [35] against the Alfort-187 strain. Neutralising antibody titres were expressed as the reciprocal dilution of serum that neutralised 100 TCID_50_ of the CSFV strains in 50% of the culture replicates.

### 2.4. PBMC and BMHC Collection and Phenotypical Analysis

PBMCs were obtained from whole blood of all the animals. Cells were separated by density-gradient centrifugation with Histopaque 1077 (Sigma-Aldrich, St. Louis, MO, USA) followed by osmotic shock in order to eliminate the remaining erythrocytes. At this time, bone marrow haematopoietic cells (BMHCs) were also obtained from the femurs and humeri of selected pigs (pigs 1, 2, 4, 5, and 6) following the protocol previously described [36,37]. The number and viability of the cells were determined by staining with Trypan Blue [38] and the cells were frozen until time of use at −80 °C in FBS with 10% dimethyl sulfoxide (DMSO).

Flow cytometry was used in order to evaluate the surface markers from PBMCs or BMHCs. Hybridoma supernatants were used for staining the swine cell surface markers CD172a (BA1C11, IgG1) [39], 6D10 (IgG2a) [20], and CD33 (5D5, IgG1) [40]. Secondary anti-Mouse IgG1 antibodies labelled with Alexa Fluor 647 or eFluor 450 (Thermofisher scientific, produced in goats and rats, respectively), as well as secondary goat Anti-Mouse IgG2a, labelled with R-Phycoerythrin (Jackson immunoresearch, West Grove, PA, USA) or Alexa Fluor 488 (Thermofisher scientific, Waltham, MA, USA), were used. Moreover, a conjugated mAb detecting CD11b from multiple species (Anti-CD11b antibody [M1/70] PE/Cy5, rat IgG2b, abcam) was also used.

For single-colour staining, cells (5 × 10^5^/well) were plated and, after removal of the medium, 50 µL of either the hybridoma supernatant or the conjugated anti-CD11b antibody were added. Following incubation at 4 °C for 20 min, the cells were washed with PBS + 2% FBS and the corresponding isotype-specific secondary antibody was added to the cells that had been incubated with hybridoma supernatant.

For two-colour staining, cells were first incubated with anti-6D10 hybridoma supernatant for 20 min at 4 °C. After washing, the second labelling was carried out either with anti-CD33 (hybridoma supernatant) or anti-CD11b (conjugated antibody). Afterwards, cells were washed with PBS + 2% FBS, followed by incubation with the isotype-specific secondary antibodies. A similar protocol was also used for three-colour staining, first labelling with the anti-6D10, followed by the anti-CD33. The anti-CD11b conjugated antibody was added when staining with the isotype-specific secondary antibodies. Finally, a viability control (propidium iodide, 1 µg/mL) was added and 30,000 live-cell events were recorded for each sample in the cytometer (FACSAriaIIu, BD Biosciences, Franklin Lakes, NJ, USA).

The cells were analyzed using the FACSDiva software, version 6.1.2, and the results were expressed as the percentage of positive cells obtained for each staining with respect to the live cells, using irrelevant isotype-matched mAbs as staining controls.

### 2.5. Sorting of 6D10+ Cells

The granulocyte precursor cell subset (6D10^+^) was sorted using a sterile magnetic sorting mechanism (Miltenyi scientific, Bergisch Gladbach, Germany). Briefly, BMHC from three Group 1 animals (CSFV Cat01 infected pigs 1, 3, and 4) and from both Group 3 animals (naïve pigs), as well as PBMC from one Group 1 animal (pig 4), were defrosted and resuspended in RPMI Medium, supplemented with 10% FBS, Pestivirus-free. Between 6–8 × 10^7^ total cells were incubated at 4 °C for 20 min with anti-6D10 at a ratio of 500 µL of hybridoma supernatant for every 1 × 10^7^ cells. The antibody was removed after centrifugation and the cells were resuspended in sorting buffer (PBS + 0.5% bovine serum albumin) containing 10% anti-mouse IgG labelled magnetic beads (Miltenyi scientific, Bergisch Gladbach, Germany) at a ratio of 100 µL of solution for every 1 × 10^7^ cells. The incubation was carried out at 4 °C for 20 min in a dark environment. After washing, the cells were resuspended in 1 mL of sorting buffer and filtered using a 70 nm filter in order to pass through the magnetic column. Afterwards, the sorted cell subsets were washed and resuspended in RPMI medium supplemented with 10% FBS.

After sorting, the cells were counted in order to determine the yield, as well as analysed by flow cytometry in order to determine the purity of the eluted fraction. In addition, the presence of CSFV RNA, as well as the viral load, in 1,000,000 cells (either pre-sorted or 6D10^+^ cell subsets) were determined by qRT-PCR [33] and by viral titration using the PLA test [27], respectively.

### 2.6. Co-Culture Experiments and Determination of IFN-γ Production by ELISPOT

The number of IFN-γ secreting cells were determined using an ELISPOT assay which was previously described [15,26,29]. Plates were coated with 5 µg/mL capture antibody (P2G10, Pharmigen, BD Biosciences, Franklin Lakes, NJ, USA). Different cell combinations were seeded in the plates and stimulated for 72 h. Thereafter IFN-γ secreting cells were revealed by sequential incubations with a biotinylated anti- IFN-γ mAb (P2C11, Pharmigen, BD Biosciences, Franklin Lakes, NJ, USA).

**Figure 1:**
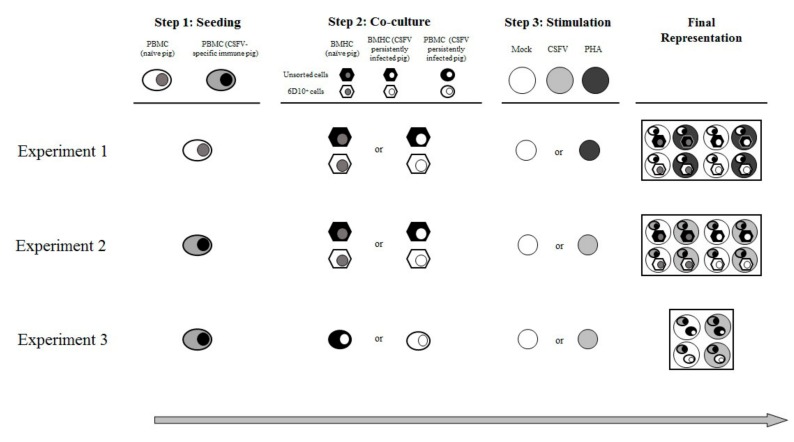
Illustration of the co-culture experiment layout. The figure shows an overview of the different cell combinations and experimental conditions tested. Three experiments were carried out, each of them consisting of three steps. Step 1: seeding, step 2: co-culture, and step 3: stimulation. A final representation of the cells and stimuli included in the wells for each experiment is also shown.

The functional assay comprised three experiments (Figure 1). For the first experiment, in step one, PBMCs from two naïve animals (pigs 10 and 11) were seeded by triplicate at a ratio of 3 × 10^5^ cells/well in a previously coated ELISPOT plate. In step two, the PBMCs were co-cultured with either unsorted BMHCs or 6D10^+^ BMHC cell subset. These cells were added at a rate of 1 × 10^5^ cells/well. The BMHCs had been collected from either naïve (pigs 10 to 12) or CSFV persistently infected animals (pigs 1 to 3). During step 3, the samples were either stimulated with phytohaemagglutinin (PHA) (10 µg/mL) or incubated in RPMI medium supplemented with 10% FBS (mock stimulated) for 72 h (Figure 1). Control wells containing only PBMCs, which were also mock or PHA stimulated, were included in the study.

For the second experiment, in step one, PBMCs, at a rate of 3 × 10^5^ cells/well, were seeded and, in step two, co-culture with the same BMHC subsets (either unsorted BMHCs or 6D10^+^ cells) from Group 3 (pigs 10 and 11) or group 1 (pigs 1, 3, and 4) at a ratio of 1 × 10^5^ cells/well was carried out. However, unlike in the previous experiment, the PBMCs used for this assay had been collected from animals showing specific CSFV cellular immune response in terms of IFN-γ production (pigs 8 and 9). The cells were either mock stimulated or stimulated with the CSFV Cat01 strain (MOI = 0.1) and were incubated for 72 h (step three). Control wells containing the PBMCs without co-culture, incubated in the presence or absence of CSFV Cat01 strain, were also included in the study (Figure 1).

For the third experiment, in the first step, PBMCs collected from an animal showing specific CSFV cellular immune response (Pig 9) were seeded at a ratio of 3 × 10^5^ cells/well. During the second step, these cells were co-cultured either with unsorted or 6D10^+^ cell subsets from pig 4 at a rate of 1 × 10^5^ cells/well, which was the same rate as the previous experiments. However, instead of BMHCs, the cell subsets used for co-culture in this experiment were sorted from PBMCs of a Group 1 animal (Pig 4). For the third step, the same stimulations (Mock or CSFV Cat01) as in the second experiment were carried out (Figure 1).

All the assays were performed three times in order to evaluate the reproducibility. After detection with the biotinylated antibody, the percentage of difference in IFN-γ production between PBMCs co-cultured with the unsorted cell population and PBMCs co-cultured with the 6D10^+^ cell subset was calculated [41].

## 3. Results

### 3.1. Establishment of CSFV Postnatal Persistent Infection

The piglets inoculated with the CSFV Cat01 strain showed fever during the first two weeks of the trial, however, the fever subsided after this time. Besides fever, these piglets did not develop other clinical signs during the first four weeks of the trial. In the last week of the trial, a variety of clinical signs were observed, such as mild and sporadic diarrhoea (pigs 2, 3, and 7), conjunctivitis (pigs 4 and 6), mild weakness of the hindquarters (pig 1), and for a few days, mild cyanosis in the ears (animals 4 and 5). Also, skin desquamation (pigs 6) and pustules were registered (pigs 4, 5, and 7).

**Figure 2:**
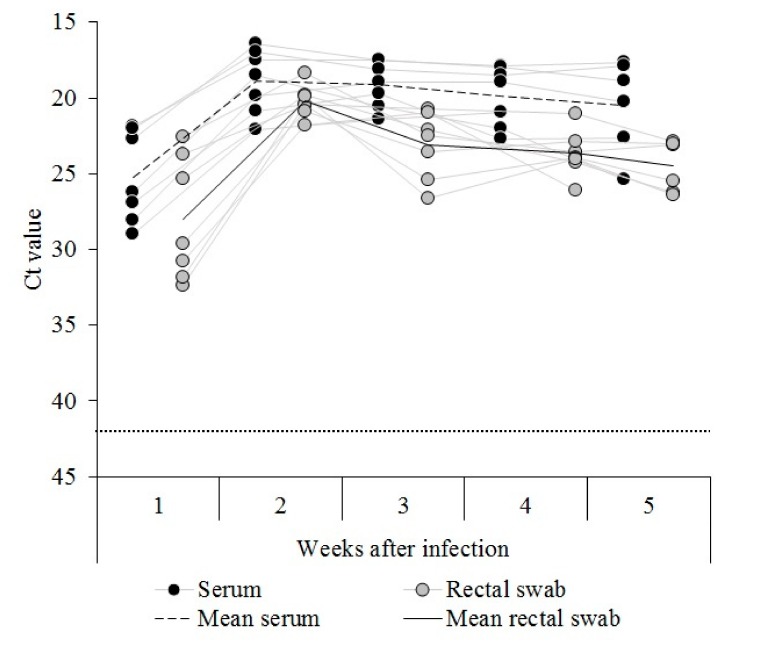
RNA detection in the sera and rectal swabs from the Cat01 infected pigs. Classical swine fever virus (CSFV) RNA was evaluated weekly in the sera (black dots) and rectal swab (gray dots) samples for 5 weeks after infection. Ct values above 42 (dotted line) were considered negative. Mean values for the serum and rectal swab samples are also indicated.

CSFV RNA was detected in sera from all the pigs inoculated with the CSFV Cat01 strain during the first week after infection. The animals showed Ct values corresponding with high (three out of seven piglets), moderate (two out of seven), and low (two out of seven) CSFV RNA load. After the second week and until the time of euthanasia, the viral RNA load detected for all infected animals was high, with the exception of pig 5, which showed moderate CSFV RNA load on the last day of the trial (Figure 2).

For the rectal swab sample, CSFV RNA load was detected in all the inoculated piglets in the first week after infection and ranged from high (one of seven piglets) to low Ct values (four out of seven animals). During the second week after infection, the CSFV RNA load increased and was high for all the animals. Thereafter, the viral RNA load in the rectal swab sample was between high and moderate for all the animals until the end of the trial (Figure 2). None of the CSFV Cat01 infected animals showed an antibody response against CSFV during the five weeks of the trial, by either the ELISA or the NPLA test.

### 3.2. Granulocyte Precursor Cells Are Increased in CSFV Persistently Infected Animals and Show a Similar Phenotype to MDSC Populations

**Figure 3:**
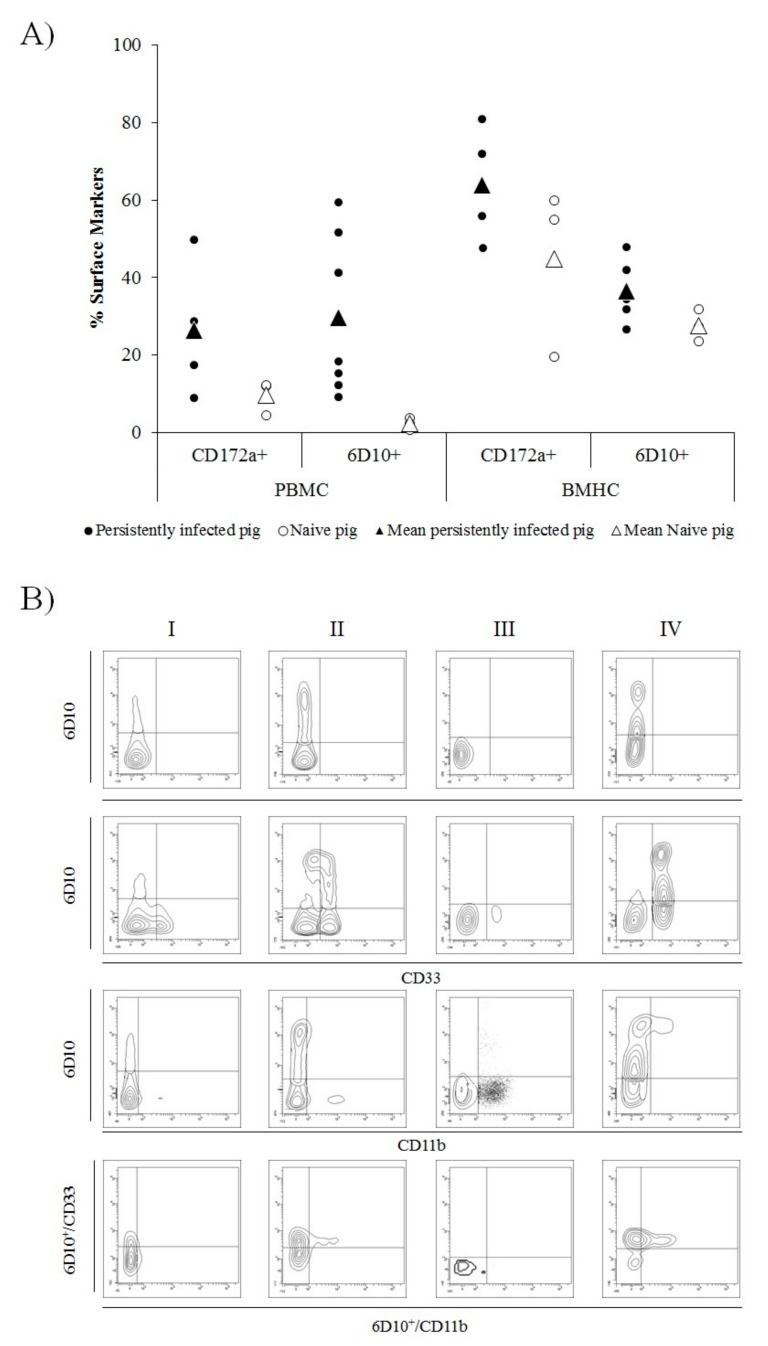
Illustration of cell surface markers in peripheral blood mononuclear cells (PBMC) and bone marrow haematopoietic cells (BMHC) from CSFV persistently infected and naïve animals at 6 weeks of age. (**A**) Comparative expression of CD172a (myelomonocytic cells) and 6D10 (granulocyte lineage) cell surface markers in PBMCs and BMHCs from CSFV persistently infected (black dots) and naïve (white dots) animals. Mean values for each group are indicated (triangle symbol). (**B**) Comparative expression of MDSC surface markers labelled by single (6D10), double (6D10/CD33 and 6D10/CD11b), and triple (6D10/CD33/CD11b) staining in different cell populations (in the bottom panels, 6D10+ cells were gated and the expression of CD33vs CD11b analysed). The cell populations evaluated were: I) BMHC from a naïve animal, II) BMHC from a CSFV persistently infected animal, III) PBMC from a naïve animal, and IV) PBMC from a CSFV persistently infected animal. The experiments were repeated twice under the same conditions.

In cells collected from CSFV persistently infected animals, between 9.17% and 59.5% of cells in the PBMC fraction were found to be 6D10^+^. In the naïve animals, by contrast, the percentage of 6D10^+^ cells in PBMC was between 0.75% and 3.85%. In the BMHC samples, the 6D10^+^ cells ranged from 26.58% to 47.9% in CSFV persistently infected piglets and from 23.7% to 31.8% in naïve animals (Figure 3A).

In the case of the CD172a cell surface marker, the percentage of CD172a^+^ cells was found to be between 8.93% and 49.84% in PBMC from CSFV persistently infected pigs. This cell subset ranged from 4.5% to 12.3% in PBMCs from naïve animals. Finally, in the BMHC samples of CSFV persistently infected pigs, between 47.8% and 80.8% of cells were CD172a^+^, whereas in the same samples from naïve animals, the percentage of CD172a+ cells ranged from 19.5% to 60% (Figure 3A).

Regarding the double and triple staining, in the BMHC samples from naïve animals, 6D10^+^, CD33^+^, and CD11b^+^ populations were found, however, only a low percentage of 6D10^+^/CD33^+^ and no 6D10^+^/CD11b^+^ double positive cells were detected (between 2.5% and 13%). In the PBMC from naïve animals, neither 6D10^+^/CD33^+^ nor 6D10^+^/CD11b^+^ were observed and, therefore, no 6D10^+^/CD33^+^/CD11b^+^ were observed. Whereas, in the BMHC samples, only between 2% and 5% of triple positive cells were found. Conversely, in BMHC and PBMC from CSFV Cat01 infected pigs, both 6D10^+^/CD33^+^ and 6D10^+^/CD11b^+^ cell populations were present, although in a lower proportion in the BMHC sample. Triple positive 6D10^+^/CD33^+^/CD11b^+^ cells were found in BMHC from persistently infected animals (representing between 6% and 12% of the 6D10^+^ cell population). Finally, the highest proportion of 6D10^+^/CD33^+^/CD11b^+^ cells was observed in PBMC samples from these animals, in which the percentage of positive cells ranged between 11.4% and 46.7% of the 6D10^+^ cell subset (Figure 3B).

### 3.3. Granulocyte Precursor Cells Are a Target for CSFV

**Figure 4:**
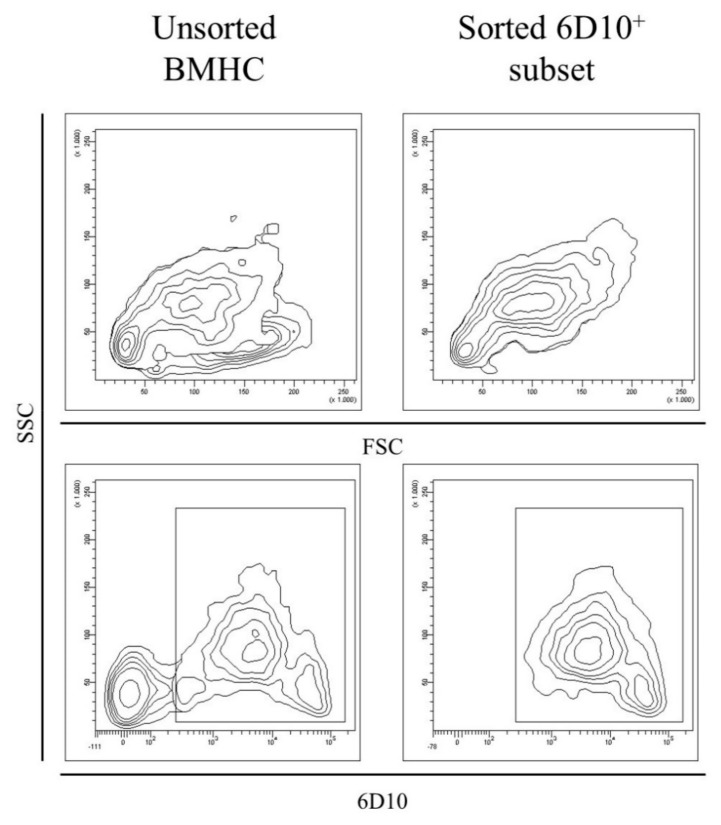
Evaluation of the unsorted and granulocytic (6D10^+^) cell populations in BMHC. The size and complexity (FSC versus SSC, upper part) as well as the expression of the 6D10 surface cell marker (lower part) was evaluated before and after magnetic sorting in order to detect differences in the phenotypic profile of BMHC and to ensure the correct performance of the magnetic sorting, respectively. The figure is representative of CSFV persistently infected animals (pig 3).

The 6D10^+^ cell subset was sorted from the BMHC of two naïve animals (pigs 10 and 11) and three CSFV persistently infected pigs (pigs 1, 3, and 4), as well as from the PBMC of pig number 4. The purity of the 6D10^+^ sorted cells was between 70% and 96% and the amount of cells recovered for each sorting ranged between 1–2 × 10^6^ cells (Figure 4). Moreover, large and small cells were observed for both the cell populations (Forward scatter (FSC), Figure 4). However, in the sorted 6D10^+^ population, the cells with the higher FSC also showed the high side scatter (SSC) values, in contrast with the unsorted cells, which showed both high and low SSC (Figure 4).

CSFV RNA was evaluated from the cell populations and the Ct value in the samples from CSFV persistently infected animals was between 22.67 and 23.5 per million cells for the whole BMHC population and between 21.84 and 22.69 per million cells for the 6D10^+^ cell subset. The viral titre was determined for these cell subsets, with both the whole BMHC cell population and the 6D10^+^ cells showing viral titres between 10^3.4^ and 10^3.95^ TCID/10^6^ cells. Finally, the viral titre was also determined for the 6D10^−^fraction in the BMHC, ranging between 10^1.6^ and 10^2,4^TCID/10^6^cells. Cells from the naïve animals were negative for CSFV RNA.

### 3.4. Granulocyte Precursor Cells from CSFV Persistently Infected Animals Are Able to Induce Immunosuppression

**Figure 5:**
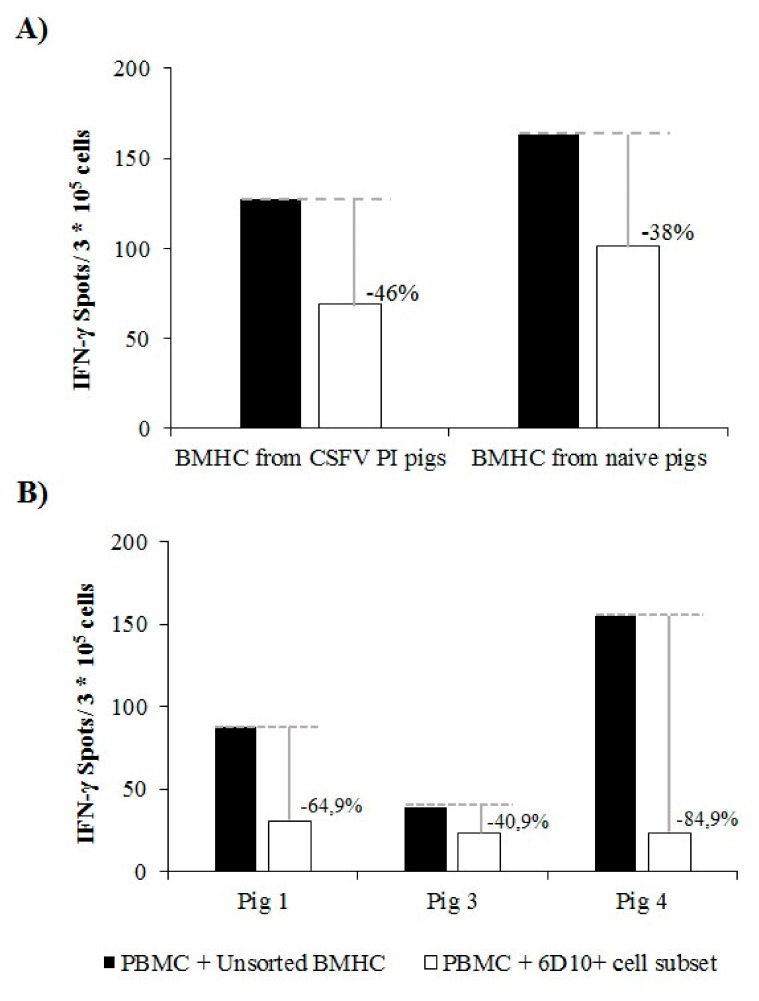
Reduction of the IFN-γ response by the granulocytic cell subset from BMHC. (**A**) PBMCs from a naïve animal were co-cultured with unsorted or 6D10^+^ cells from the BMHC of naïve or CSFV persistently infected pigs in a 1:3 ratio and stimulated with phytohaemagglutinin (PHA). (**B**) PBMCs from a CSFV-specific immune animal were co-cultured with unsorted or 6D10^+^ cells from BMHC of naïve or CSFV persistently infected pigs in a 1:3 ratio and stimulated with CSFV Cat01 (MOI = 0.1). The x axis indicates the origin of the BMHCs used for co-culture. IFN-γ production was measured by ELISPOT assay. All the assays were performed by triplicate to corroborate the results.

PBMCs from naïve animals showed a normal response in terms of IFN-γ when stimulated with PHA in the absence of any co-culture (200–250 IFN-γ spots/ 3 × 10^5^ cells). The mean IFN-γ production after PHA stimulation in PBMCs from naïve animals was 150 and 90 IFN-γ spots/ 3 × 10^5^ cells when these cells were co-cultured in the presence of the whole BMHC or 6D10^+^ cell populations from naïve animals, respectively (reduction of 38%, Figure 5A). A slightly higher reduction in the IFN-γ response was observed after co-culture of the same PBMC from naïve animals with the different BMHC cell subsets from CSFV PI animals. In this regard, the IFN-γ response was reduced by 46% when the cells were co-cultured with the 6D10^+^subset compared to the co-culture and stimulation with the whole BMHC population. Specifically, around 130 and 70 IFN-γ spots/ 3 × 10^5^ cells were evidenced for PBMCs co-cultured with the unsorted BMHC and the 6D10^+^ cell subsets, respectively (Figure 5A).

With regard to the second functional experiment, no differences in the CSFV specific IFN-γ response were observed between the co-culture of PBMCs from vaccinated animals with either the unsorted or 6D10^+^ cell subsets of BMHC from naïve animals. When the PBMCs from vaccinated animals were co-cultured with the unsorted BMHC population from CSFV persistently infected pigs, the IFN-γ response ranged between 39 and 155 spots/ 3 × 10^5^ PBMC (Figure 5B). However, the IFN-γ levels observed when PBMCs were co-cultured with the 6D10^+^ cell population and stimulated with CSFV were between 24 and 30 spots/ 3 × 10^5^ cells (Figure 5B), thus showing a reduction in the IFN-γ response between 40.9% and 84.9%.

**Figure 6:**
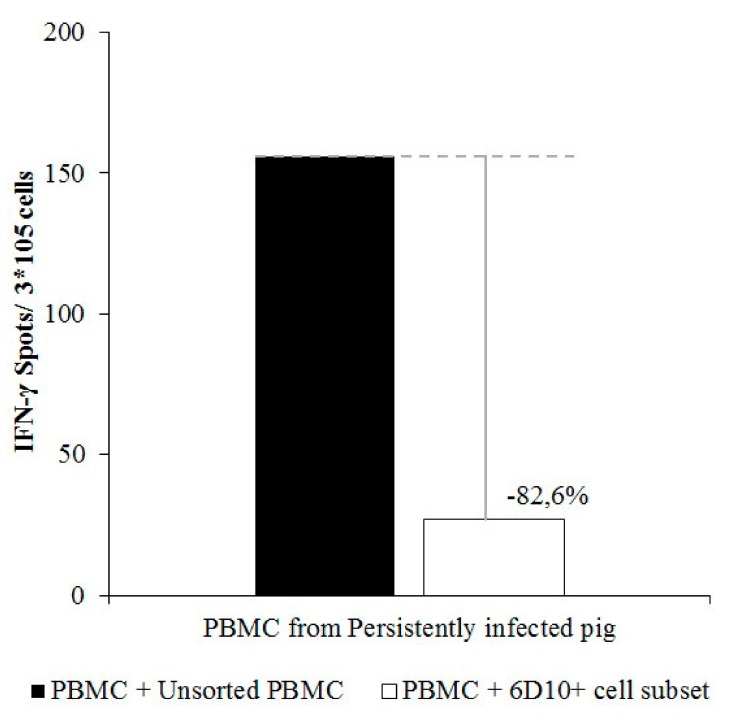
Reduction of the IFN-γ response by the granulocytic cell subset from PBMC of CSFV persistently infected animals. PBMCs from a CSFV-specific immune animal were co-cultured with unsorted (black bars) or sorted 6D10^+^ (white bars) cells from PBMC of a CSFV persistently infected pig in a 1:3 ratio and stimulated with CSFV Cat01 (MOI = 0.1). The x axis indicates the origin of the PBMCs used for co-culture. IFN-γ production was measured by ELISPOT assay. All the assays were performed by triplicate to corroborate the results.

Finally, the cellular immune response, in terms of IFN-γ, in PBMC from a vaccinated animal that had previously shown specific IFN-γ response against CSFV (Pig 9) was altered when these cells were stimulated in the presence of different PBMC populations from animal 4 (CSFV persistently infected animal). In this regard, IFN-γ production after CSFV stimulation was determined to be 156 spots/ 3 × 10^5^cells when PBMCs from pig 9 were co-cultured in the presence of the full PBMC population from pig 4. However, when PBMCs from pig 9 were stimulated with CSFV in the presence of the 6D10^+^ cell subset from pig 4, IFN-γ production was around 27 spots/ 3 × 10^5^cells, showing a strong reduction of 82.9% (Figure 6).

## 4. Discussion

The capacity of the viruses from the *Pestivirus* genus to induce persistent infection is one of their most striking features, being fundamental for the maintenance of these viruses in farms. In this regard, the CSFV ability to induce persistence after a congenital infection has been documented since the late 1970s [11,12]. However, more recent studies have also proven the establishment of CSFV persistent infection following postnatal infection in both domestic pigs and wild boar [15,32]. Nevertheless, the role of viral factors, such as the virulence or genogroup of the infecting strain, in the establishment of this form of disease remains to be studied. Additionally, the immunological phenomena taking place, as well as the alterations in the phenotypic profile of cells from the immune system in persistently infected pigs have not been fully elucidated. The animals infected with the Cat01 strain in the present study did not develop any humoral response against the virus and were found to be highly viraemic and shedding high amounts of the virus throughout the study. Therefore, these animals complied with the criteria for persistently infected animals previously stablished [6,7].

Regarding the changes in cell populations, the increase in the percentage of 6D10^+^ cells in CSFV persistently infected animals after five weeks post infection is remarkable. This surface marker is present in cells of the granulocytic lineage and is highly expressed in precursor forms [20]. Additionally, 6D10^+^/CD33^+^/CD11b^+^ cells were evidenced in BMHC and particularly PBMC from persistently infected animals, whereas they were not found in the naïve pigs. Taking into account that MDSC are immature cell populations, as well as their characterization in other species as CD33^+^/CD11b^+^ [23,24,42], our findings strongly suggested the role of an MDSC population in CSFV persistently infected animals. This cell population most likely represents PMN-MDSC, considering their granulocytic lineage, as well as their high size and complexity. Notably, it has been reported that, in the majority of cancer models where MDSC have been characterized, PMN-MDSC represent more than 80% of all MDSC [43,44]. The variability in the percentage of 6D10^+^ cells in PBMC and BMHC between the persistently infected animals from the present study might be attributed to factors such as the natural variability of each individual or even the fact that PMN-MDSC have been reported as being particularly sensitive to cryopreservation and/or thawing [45].

It should be noted that the surface markers evaluated in the present work could also be present in mature neutrophils. However, PMN-MDSC are low density cells and thus would remain in the upper fraction during the density gradient separation by which PBMCs are collected, whereas neutrophils would likely be lost during this process [46]. In addition, the 6D10^+^ cell subset from CSFV persistently infected pigs was found to have immunosuppressive capabilities, reducing the IFN-γ production by PBMC after PHA stimulation, an important feature of MDSC characterization [21,23]. Most notably, this cell subset also induced suppression in terms of IFN-γ production of antigen-specific PBMC responses to CSFV. This finding is particularly significant given that this type of assay has been described as more relevant to in vivo conditions [23]. The largest reduction in the IFN-γ production was observed in the antigen-specific assays, which is in agreement with previous studies, which have shown that a suppression in antigen-specific immune response is a primary feature of PMN-MDSC, in contrast to M-MDSC [47,48].

It is worth highlighting that the increase of the 6D10^+^ cell population was found in both BMHC and PBMC from persistently infected pigs. This implies that these cells are actively circulating in the bloodstream of these animals. Studies of persistent infection in humans in which MDSC have been implicated, such as HIV and HCV, have reported a similar increase of MDSC in PBMC from persistently infected patients [4,5]. The generation of MDSC populations during persistent infection has been associated with abnormal myelopoiesis and activation of neutrophils and monocytes. In normal conditions, this activation does not last long, as the stimulus is eliminated from the organism. Conversely, a prolonged stimulation might lead to pathological activation of myelopoiesis, which will produce cells with altered functions such as poor phagocytosis and production of anti-inflammatory cytokines [23,24,49]. MDSC use multiple mechanisms to induce immunosuppression in the host, mainly IL-10, iNOS, and reactive oxygen species. The production of high levels of IL-10 by PBMC from CSFV persistently infected pigs has been previously reported [15], further suggesting the role of MDSC populations in this form of infection. However, the blocking of IL-10 did not restore the production of IFN-γ in these cells, implying that there are other mechanisms involved in the immune suppression detected in these animals. Hence, the specific biochemical pathways used by the 6D10^+^ cells from CSFV persistently infected animals to induce immune suppression remain to be elucidated and should be the subject of future works. Additionally, the presence of MDSC populations in organs from CSFV persistently infected animals cannot be discarded and warrants further studies considering that they have also been found in tumour microenvironments as well as in other lymphoid organs in cancer patients [43,44].

Previous results have shown that CSFV persistent infection can be established when animals are infected as late as 21 days after birth [16], although at a lower rate than when pigs are infected a few hours after birth. This finding suggests a role of age in the establishment of CSFV persistent infection, which is likely related to the presence of MDSC. Previous studies showed that this cell population has been found in cord blood and around the neonatal period in humans and it has been implied that their presence might contribute to an increased susceptibility to infection [41]. So far, other aspects such as gender or swine leukocyte antigen type have not been related to MDSC.

Taken together, our results suggest that MDSC are playing a role in the establishment and maintenance of CSFV persistent infection in swine. The cells characterized in the present work showed a similar phenotype to previously described human MDSC populations, although no equivalent has been described for the 6D10^+^ surface marker in humans [20]. The fact that only a single marker (6D10^+^) was used for the sorting of a cell subset, which was found to have immunosuppressive capabilities, may facilitate the study of MDSC in swine. Nevertheless, a thorough characterization of the mechanisms used by MDSC to generate persistent infection requires further studies. The information obtained by studying the role of MDSC during CSFV persistent infection may extrapolate to other viral persistent infections in mammals where similar immunosuppressive mechanisms are taking place such as HIV, HCV, or HBV among others. In this manner, useful insight into the functionality of MDSC could be achieved, which is relevant for a thorough understanding of viral persistence, and may have repercussions in viral therapy and disease control.
